# Supplementary material for: Assessing COVID-19 Infection and Severe Disease Risk in Cancer Patients and Survivors: The Role of Vaccination Status, Circulating Variants, and Comorbidities—A Population-Based Study in Northern Italy
Source: Vaccines (Basel). 2025 Dec 3;13(12):1223. doi: 10.3390/vaccines13121223 (PMC12737404; doi:10.3390/vaccines13121223)
Supplement: Supplementary file 1 [file vaccines-13-01223-s001.zip › vaccines-4003044-supplementary.pdf]

**Table S1**. Proportion of variants in the transition period.

| Date of Survey | Variant | % |
|---|---|---|
| 18-ott-21 | Delta | 100,0% |
| 04-nov-21 | Delta | 100,0% |
| 18-nov-21 | Delta | 100,0% |
| 25-nov-21 | Delta | 100,0% |
| 06-dic-21 | Delta | 68,6% |
| | Omicron | 31,4% |
| 05-gen-22 | Delta | 23,0% |
| | Omicron | 77,0% |
| 20-gen-22 | Delta | 10,0% |
| | Omicron | 90,0% |
| 02-feb-22 | Delta | 0,0% |
| | Omicron | 100,0% |
| 28-feb-22 | Delta | 0,0% |
| | Omicron | 100,0% |

**Table S2.** Cohort characteristics at January 1, 2020 by history of cancer, Reggio Emilia province, Italy.

| | Population without cancer | Population with cancer | |
|---|---|---|---|
| | | Incidence 1996-2019 | Incidence 2020-2021 |
| **Overall** | **505,209** | **26,928** | **6,379** |
| **Age group** | | | |
| 0-4 | 21,233 | 10 | 8 |
| 5-17 | 70,333 | 113 | 34 |
| 18-34 | 93,052 | 536 | 132 |
| 35-64 | 224,994 | 8,958 | 2,354 |
| 65-79 | 65,253 | 10,657 | 2,567 |
| 80+ | 30,344 | 6,654 | 1,284 |
| **Sex** | | | |
| M | 250,202 | 12,067 | 3,127 |
| F | 255,007 | 14,861 | 3,252 |
| **Charlson Comorbidity Index** | | | |
| 0 | 486,213 | 21,777 | 5,608 |
| 1 | 12,998 | 2,443 | 473 |
| 2 | 3,986 | 820 | 165 |
| 3 | 2,012 | 1,888 | 133 |

**Table S3**. Risk of SARS-CoV-2 infection by cancer prevalence and immunisation status adjusted for sex, age and Charlson Comorbidity Index in the pre-Omicron and Omicron BA.1 periods, Reggio Emilia province, Italy, 20 February 2020–30 September 2022.

| | | **Risk of SARS-CoV-2 infection** | | | | | | | | | |
|---|---|---|---|---|---|---|---|---|---|---|---|
| | | **Period of infection from Feb 20, 2020 to Dec 20, 2021** | | | | | **Period of infection from Jan 01, 2022 to Sep 30, 2022** | | | | |
| | | **Persons-days** | **Infections** | **HR** | **95% IC** | | **Persons-days** | **Infections** | **HR** | **95% IC** | |
| **No infection, no vaccine** | | | | | | | | | | | |
| HR for pop no cancer | | 251,912,448 | 46,243 | 1 | | | 17,809,840 | 22,462 | 1 | | |
| HR for pop with cancer | | 11,841,178 | 2,210 | 1.02 | 0.97 | 1.06 | 321,217 | 435 | 1.64 | 1.49 | 1.80 |
| | <2 | 1,504,078 | 429 | 1.40 | 1.27 | 1.54 | 25,634 | 70 | 2.59 | 2.06 | 3.25 |
| | 2-5 | 2,540,316 | 470 | 0.96 | 0.88 | 1.06 | 71,183 | 101 | 1.73 | 1.43 | 2.11 |
| | >5 | 7,796,784 | 1,311 | 0.94 | 0.89 | 1.00 | 224,400 | 264 | 1.47 | 1.30 | 1.65 |
| **No infection, 1 dose** | | | | | | | | | | | |
| HR for pop no cancer | | 14,630,632 | 1,488 | 0.71 | 0.67 | 0.75 | 1,094,708 | 2,753 | 1.15 | 1.11 | 1.20 |
| HR for pop with cancer | | 918,722 | 80 | 0.47 | 0.37 | 0.58 | 23,883 | 36 | 1.18 | 0.86 | 1.64 |
| | <2 | 141,877 | 16 | 0.60 | 0.37 | 0.99 | 4,813 | 9 | 1.41 | 0.73 | 2.71 |
| | 2-5 | 171,583 | 14 | 0.41 | 0.24 | 0.69 | 4,147 | 8 | 1.52 | 0.78 | 2.97 |
| | >5 | 605,262 | 50 | 0.45 | 0.34 | 0.60 | 14,923 | 19 | 1.01 | 0.65 | 1.58 |
| **No infection, 2 doses** | | | | | | | | | | | |
| HR for pop no cancer | | 53,025,309 | 4,192 | 0.36 | 0.35 | 0.37 | 14,500,761 | 31,051 | 1.32 | 1.30 | 1.34 |
| HR for pop with cancer | | 4,736,273 | 257 | 0.33 | 0.29 | 0.37 | 331,742 | 588 | 1.51 | 1.39 | 1.64 |
| | <2 | 690,189 | 46 | 0.41 | 0.31 | 0.55 | 64,425 | 143 | 1.70 | 1.45 | 2.01 |
| | 2-5 | 979,014 | 58 | 0.36 | 0.28 | 0.46 | 60,692 | 89 | 1.24 | 1.01 | 1.53 |
| | >5 | 3,067,070 | 153 | 0.30 | 0.26 | 0.35 | 206,625 | 356 | 1.53 | 1.37 | 1.70 |
| **No infection, 3 or more doses** | | | | | | | | | | | |
| HR for pop no cancer | | 2,708,425 | 228 | 0.15 | 0.13 | 0.17 | 65,025,072 | 61,709 | 1.04 | 1.03 | 1.06 |
| HR for pop with cancer | | 567,032 | 34 | 0.11 | 0.08 | 0.15 | 5,571,101 | 5,059 | 1.29 | 1.24 | 1.34 |
| | <2 | 75,587 | 8 | 0.21 | 0.10 | 0.42 | 648,840 | 759 | 1.56 | 1.45 | 1.68 |
| | 2-5 | 119,224 | 8 | 0.12 | 0.06 | 0.23 | 1,126,850 | 1,105 | 1.35 | 1.27 | 1.44 |
| | >5 | 372,221 | 18 | 0.09 | 0.06 | 0.14 | 3,795,411 | 3,195 | 1.21 | 1.16 | 1.27 |
| **Infection, no vaccine** | | | | | | | | | | | |
| HR for pop no cancer | | 5,521,098 | 127 | 0.10 | 0.09 | 0.12 | 4,970,897 | 3,902 | 1.10 | 1.06 | 1.14 |
| HR for pop with cancer | | 222,370 | 8 | 0.15 | 0.07 | 0.30 | 88,452 | 73 | 1.79 | 1.43 | 2.24 |
| | <2 | 37,821 | 1 | 0.11 | 0.02 | 0.80 | 12,696 | 16 | 2.48 | 1.56 | 3.95 |
| | 2-5 | 46,579 | 3 | 0.25 | 0.08 | 0.77 | 18,195 | 17 | 2.07 | 1.31 | 3.27 |
| | >5 | 137,970 | 4 | 0.12 | 0.05 | 0.33 | 57,561 | 40 | 1.53 | 1.13 | 2.08 |
| **Infection, 1 dose** | | | | | | | | | | | |
| HR for pop no cancer | | 3,739,067 | 22 | 0.03 | 0.02 | 0.04 | 1,765,645 | 1,477 | 0.60 | 0.57 | 0.63 |
| HR for pop with cancer | | 228,024 | 1 | 0.02 | 0.00 | 0.17 | 32,247 | 34 | 0.76 | 0.55 | 1.06 |
| | <2 | 31,728 | 0 | - | - | - | 5,115 | 6 | 0.80 | 0.38 | 1.69 |
| | 2-5 | 54,258 | 0 | - | - | - | 5,835 | 7 | 0.77 | 0.37 | 1.61 |
| | >5 | 142,038 | 1 | 0.04 | 0.01 | 0.28 | 21,297 | 21 | 0.75 | 0.49 | 1.14 |
| **Infection, 2 doses** | | | | | | | | | | | |
| HR for pop no cancer | | 1,898,408 | 14 | 0.03 | 0.02 | 0.05 | 10,531,276 | 3,691 | 0.52 | 0.50 | 0.54 |
| HR for pop with cancer | | 141,443 | 1 | 0.03 | 0.00 | 0.20 | 352,240 | 159 | 0.70 | 0.60 | 0.82 |
| | <2 | 25,339 | 0 | - | - | - | 46,563 | 21 | 0.69 | 0.45 | 1.04 |
| | 2-5 | 28,187 | 0 | - | - | - | 79,741 | 35 | 0.66 | 0.48 | 0.92 |
| | >5 | 87,917 | 1 | 0.04 | 0.01 | 0.32 | 225,936 | 103 | 0.72 | 0.60 | 0.87 |
| **Infection, 3 or more doses** | | | | | | | | | | | |
| HR for pop no cancer | | 62,788 | 1 | 0.02 | 0.00 | 0.17 | 7,672,983 | 2,353 | 0.64 | 0.62 | 0.67 |
| HR for pop with cancer | | 10,716 | 0 | - | - | - | 566,147 | 160 | 0.80 | 0.69 | 0.93 |
| | <2 | 1,977 | 0 | - | - | - | 71,946 | 29 | 1.01 | 0.71 | 1.44 |
| | 2-5 | 2,497 | 0 | - | - | - | 131,307 | 32 | 0.71 | 0.51 | 1.00 |
| | >5 | 6,242 | 0 | - | - | - | 362,894 | 99 | 0.79 | 0.65 | 0.96 |

**Table S4**. Risk of severe disease and death from COVID-19 by cancer prevalence and immunisation status adjusted for sex, age and Charlson Comorbidity Index in the pre-Omicron and Omicron BA.1 periods, Reggio Emilia province, Italy, 20 February 2020–30 September 2022.

| | | Risk of severe disease and death from COVID-19 | | | | | | | | | |
|---|---|---|---|---|---|---|---|---|---|---|---|
| | | Period of infection from Feb 20, 2020 to Dec 20, 2021 | | | | | Period of infection from Jan 01, 2022 to Sep 30, 2022 | | | | |
| | | n | Events | OR | 95% IC | | n | Events | OR | 95% IC | |
| **No infection, no vaccine** | | | | | | | | | | | |
| HR for pop no cancer | | 46,243 | 4,092 | 1 | | | 22,462 | 246 | 1 | | |
| HR for pop with cancer | | 2,210 | 665 | 1.27 | 1.14 | 1.42 | 435 | 31 | 1.15 | 0.75 | 1.77 |
| | <2 | 429 | 153 | 1.83 | 1.46 | 2.30 | 70 | 4 | 1.30 | 0.45 | 3.77 |
| | 2-5 | 470 | 128 | 1.26 | 1.00 | 1.60 | 101 | 8 | 1.32 | 0.57 | 3.06 |
| | >5 | 1,311 | 384 | 1.11 | 0.97 | 1.28 | 264 | 19 | 1.04 | 0.61 | 1.78 |
| **No infection, 1 dose** | | | | | | | | | | | |
| HR for pop no cancer | | 1,488 | 119 | 0.54 | 0.44 | 0.67 | 2,753 | 30 | 1.20 | 0.80 | 1.80 |
| HR for pop with cancer | | 80 | 30 | 1.29 | 0.78 | 2.13 | 36 | 2 | 0.83 | 0.17 | 4.14 |
| | <2 | 16 | 6 | 1.74 | 0.59 | 5.15 | 9 | 0 | - | - | - |
| | 2-5 | 14 | 5 | 1.46 | 0.41 | 5.16 | 8 | 0 | - | - | - |
| | >5 | 50 | 19 | 1.14 | 0.61 | 2.14 | 19 | 2 | 1.58 | 0.29 | 8.71 |
| **No infection, 2 doses** | | | | | | | | | | | |
| HR for pop no cancer | | 4,192 | 164 | 0.29 | 0.24 | 0.34 | 31,051 | 157 | 0.36 | 0.29 | 0.45 |
| HR for pop with cancer | | 257 | 46 | 0.61 | 0.43 | 0.86 | 588 | 38 | 1.09 | 0.75 | 1.59 |
| | <2 | 46 | 11 | 1.11 | 0.52 | 2.37 | 143 | 18 | 3.11 | 1.79 | 5.42 |
| | 2-5 | 58 | 7 | 0.39 | 0.16 | 0.91 | 89 | 4 | 0.58 | 0.19 | 1.75 |
| | >5 | 153 | 28 | 0.58 | 0.37 | 0.90 | 356 | 16 | 0.66 | 0.38 | 1.13 |
| **No infection, 3 or more doses** | | | | | | | | | | | |
| HR for pop no cancer | | 228 | 21 | 0.35 | 0.22 | 0.56 | 61,709 | 821 | 0.24 | 0.21 | 0.29 |
| HR for pop with cancer | | 34 | 6 | 0.55 | 0.21 | 1.41 | 5,059 | 288 | 0.43 | 0.35 | 0.54 |
| | <2 | 8 | 1 | 0.47 | 0.05 | 4.28 | 759 | 57 | 0.80 | 0.58 | 1.12 |
| | 2-5 | 8 | 2 | 0.71 | 0.13 | 3.86 | 1,105 | 60 | 0.47 | 0.34 | 0.66 |
| | >5 | 18 | 3 | 0.50 | 0.13 | 1.90 | 3,195 | 171 | 0.35 | 0.27 | 0.45 |
| **Infection, no vaccine** | | | | | | | | | | | |
| HR for pop no cancer | | 127 | 10 | 0.78 | 0.37 | 1.67 | 3,902 | 15 | 0.34 | 0.20 | 0.59 |
| HR for pop with cancer | | 8 | 2 | 0.38 | 0.07 | 2.18 | 73 | 1 | 0.32 | 0.04 | 2.42 |
| | <2 | 1 | 0 | - | - | - | 16 | 0 | - | - | - |
| | 2-5 | 3 | 0 | - | - | - | 17 | 0 | - | - | - |
| | >5 | 4 | 2 | 1.41 | 0.17 | 11.39 | 40 | 1 | 0.74 | 0.10 | 5.60 |
| **Infection, 1 dose** | | | | | | | | | | | |
| HR for pop no cancer | | 22 | 1 | 0.56 | 0.06 | 5.42 | 1,477 | 9 | 0.55 | 0.28 | 1.10 |
| HR for pop with cancer | | 1 | 0 | - | - | - | 34 | 0 | - | - | - |
| | <2 | 0 | 0 | - | - | - | 6 | 0 | - | - | - |
| | 2-5 | 0 | 0 | - | - | - | 7 | 0 | - | - | - |
| | >5 | 1 | 0 | - | - | - | 21 | 0 | - | - | - |
| **Infection, 2 doses** | | | | | | | | | | | |
| HR for pop no cancer | | 14 | 0 | - | - | - | 3,691 | 28 | 0.24 | 0.16 | 0.37 |
| HR for pop with cancer | | 1 | 0 | - | - | - | 159 | 9 | 0.51 | 0.24 | 1.10 |
| | <2 | 0 | 0 | - | - | - | 21 | 2 | 1.26 | 0.26 | 6.24 |
| | 2-5 | 0 | 0 | - | - | - | 35 | 1 | 0.43 | 0.05 | 3.61 |
| | >5 | 1 | 0 | - | - | - | 103 | 6 | 0.42 | 0.16 | 1.08 |
| **Infection, 3 or more doses** | | | | | | | | | | | |
| HR for pop no cancer | | 1 | 0 | - | - | - | 2,353 | 24 | 0.16 | 0.10 | 0.26 |
| HR for pop with cancer | | 0 | 0 | - | - | - | 160 | 5 | 0.22 | 0.08 | 0.59 |
| | <2 | 0 | 0 | - | - | - | 29 | 4 | 2.27 | 0.71 | 7.30 |
| | 2-5 | 0 | 0 | - | - | - | 32 | 0 | - | - | - |
| | >5 | 0 | 0 | - | - | - | 99 | 1 | 0.05 | 0.01 | 0.35 |

**Table S5**. Sensitivity analysis of risk of SARS-CoV-2 infection by cancer prevalence and immunisation status adjusted for sex, age and Charlson Comorbidity Index in the pre-Omicron and Omicron BA.1 periods, Reggio Emilia province, Italy, 20 February 2020–30 September 2022, considering a 14-day lag time from vaccine dose administration and protection for the first and second dose.

| | | Risk of SARS-CoV-2 infection | | | | | | | | | |
|---|---|---|---|---|---|---|---|---|---|---|---|
| | | Period of infection from Feb 20, 2020 to Dec 20, 2021 | | | | | Period of infection from Jan 01, 2022 to Sep 30, 2022 | | | | |
| | | Persons-days | Infections | HR | 95% IC | | Persons-days | Infections | HR | 95% IC | |
| **No infection, no vaccine** | | | | | | | | | | | |
| HR for pop no cancer | | 256,861,090 | 46,780 | 1 | | | 18,055,416 | 23,449 | 1 | | |
| HR for pop with cancer | | 12,188,166 | 2,250 | 1.01 | 0.96 | 1.06 | 324,212 | 441 | 1.62 | 1.47 | 1.78 |
| | <2 | 1,533,932 | 435 | 1.39 | 1.27 | 1.53 | 26,266 | 71 | 2.48 | 1.98 | 3.11 |
| | 2-5 | 2,611,751 | 478 | 0.96 | 0.87 | 1.05 | 71,677 | 103 | 1.73 | 1.43 | 2.10 |
| | >5 | 8,042,483 | 1,337 | 0.93 | 0.88 | 0.99 | 226,269 | 267 | 1.45 | 1.29 | 1.63 |
| **No infection, 1 dose** | | | | | | | | | | | |
| HR for pop no cancer | | 14,468,682 | 1,172 | 0.58 | 0.55 | 0.62 | 1,154,818 | 2,281 | 1.00 | 0.96 | 1.05 |
| HR for pop with cancer | | 917,391 | 60 | 0.46 | 0.36 | 0.60 | 25,674 | 37 | 1.17 | 0.85 | 1.62 |
| | <2 | 141,891 | 16 | 0.78 | 0.48 | 1.28 | 5,243 | 10 | 1.47 | 0.79 | 2.73 |
| | 2-5 | 171,480 | 10 | 0.39 | 0.21 | 0.73 | 4,406 | 7 | 1.30 | 0.62 | 2.74 |
| | >5 | 604,020 | 34 | 0.40 | 0.29 | 0.57 | 16,025 | 20 | 1.03 | 0.67 | 1.60 |
| **No infection, 2 doses** | | | | | | | | | | | |
| HR for pop no cancer | | 48,238,630 | 3,971 | 0.37 | 0.36 | 0.39 | 14,195,075 | 30,536 | 1.35 | 1.32 | 1.37 |
| HR for pop with cancer | | 4,390,616 | 237 | 0.33 | 0.29 | 0.38 | 326,956 | 581 | 1.54 | 1.41 | 1.67 |
| | <2 | 643,915 | 40 | 0.39 | 0.29 | 0.54 | 63,489 | 141 | 1.73 | 1.46 | 2.04 |
| | 2-5 | 906,885 | 54 | 0.37 | 0.28 | 0.48 | 59,904 | 88 | 1.26 | 1.02 | 1.56 |
| | >5 | 2,839,816 | 143 | 0.31 | 0.26 | 0.37 | 203,563 | 352 | 1.55 | 1.40 | 1.73 |
| **No infection, 3 or more doses** | | | | | | | | | | | |
| HR for pop no cancer | | 2,708,412 | 228 | 0.15 | 0.13 | 0.17 | 65,025,072 | 61,709 | 1.03 | 1.01 | 1.05 |
| HR for pop with cancer | | 567,032 | 34 | 0.11 | 0.08 | 0.16 | 5,571,101 | 5,059 | 1.29 | 1.24 | 1.34 |
| | <2 | 75,587 | 8 | 0.21 | 0.11 | 0.43 | 648,840 | 759 | 1.57 | 1.46 | 1.68 |
| | 2-5 | 119,224 | 8 | 0.12 | 0.06 | 0.24 | 1,126,850 | 1,105 | 1.35 | 1.27 | 1.44 |
| | >5 | 372,221 | 18 | 0.09 | 0.06 | 0.14 | 3,795,411 | 3,195 | 1.22 | 1.16 | 1.27 |
| **Infection, no vaccine** | | | | | | | | | | | |
| HR for pop no cancer | | 5,979,809 | 128 | 0.10 | 0.08 | 0.12 | 5,015,744 | 3,923 | 1.05 | 1.02 | 1.09 |
| HR for pop with cancer | | 246,490 | 8 | 0.14 | 0.07 | 0.28 | 88,871 | 74 | 1.78 | 1.42 | 2.22 |
| | <2 | 42,166 | 1 | 0.11 | 0.01 | 0.75 | 12,785 | 16 | 2.40 | 1.51 | 3.81 |
| | 2-5 | 51,944 | 3 | 0.23 | 0.08 | 0.73 | 18,251 | 17 | 2.04 | 1.29 | 3.23 |
| | >5 | 152,380 | 4 | 0.11 | 0.04 | 0.31 | 57,835 | 41 | 1.54 | 1.14 | 2.08 |
| **Infection, 1 dose** | | | | | | | | | | | |
| HR for pop no cancer | | 3,505,986 | 21 | 0.03 | 0.02 | 0.04 | 1,926,909 | 1,527 | 0.53 | 0.50 | 0.56 |
| HR for pop with cancer | | 220,570 | 1 | 0.02 | 0.00 | 0.17 | 38,274 | 34 | 0.59 | 0.42 | 0.82 |
| | <2 | 30,216 | 0 | - | - | - | 6,174 | 6 | 0.61 | 0.28 | 1.30 |
| | 2-5 | 52,567 | 0 | - | - | - | 7,229 | 8 | 0.66 | 0.33 | 1.32 |
| | >5 | 137,787 | 1 | 0.04 | 0.01 | 0.27 | 24,871 | 20 | 0.56 | 0.36 | 0.87 |
| **Infection, 2 doses** | | | | | | | | | | | |
| HR for pop no cancer | | 1,672,778 | 14 | 0.03 | 0.02 | 0.06 | 10,325,165 | 3,620 | 0.56 | 0.54 | 0.58 |
| HR for pop with cancer | | 124,777 | 1 | 0.04 | 0.01 | 0.26 | 345,794 | 158 | 0.75 | 0.64 | 0.87 |
| | <2 | 23,003 | 0 | - | - | - | 45,690 | 21 | 0.74 | 0.49 | 1.12 |
| | 2-5 | 24,382 | 0 | - | - | - | 78,373 | 34 | 0.68 | 0.49 | 0.95 |
| | >5 | 77,392 | 1 | 0.06 | 0.01 | 0.41 | 221,731 | 103 | 0.77 | 0.64 | 0.94 |
| **Infection, 3 or more doses** | | | | | | | | | | | |
| HR for pop no cancer | | 62,788 | 1 | 0.02 | 0.00 | 0.18 | 7,672,983 | 2,353 | 0.65 | 0.62 | 0.67 |
| HR for pop with cancer | | 10,716 | 0 | - | - | - | 566,147 | 160 | 0.80 | 0.69 | 0.93 |
| | <2 | 1,977 | 0 | - | - | - | 71,946 | 29 | 1.01 | 0.71 | 1.44 |
| | 2-5 | 2,497 | 0 | - | - | - | 131,307 | 32 | 0.71 | 0.51 | 1.00 |
| | >5 | 6,242 | 0 | - | - | - | 362,894 | 99 | 0.79 | 0.65 | 0.96 |

**Table S6**. Sensitivity analysis of risk of severe disease and death from COVID-19 by cancer prevalence and immunisation status adjusted for sex, age and Charlson Comorbidity Index in the pre-Omicron and Omicron BA.1 periods, Reggio Emilia province, Italy, 20 February 2020–30 September 2022, considering a 14-day lag time from vaccine dose administration and protection for the first and second dose.

| | | Risk of severe disease and death from COVID-19 | | | | | | | | | |
|---|---|---|---|---|---|---|---|---|---|---|---|
| | | Period of infection from Feb 20, 2020 to Dec 20, 2021 | | | | | Period of infection from Jan 01, 2022 to Sep 30, 2022 | | | | |
| | | **n** | **Events** | **OR** | **95% IC** | | **n** | **Events** | **OR** | **95% IC** | |
| **No infection, no vaccine** | | | | | | | | | | | |
| HR for pop no cancer | | 46,780 | 4,161 | 1 | | | 23,449 | 253 | 1 | | |
| HR for pop with cancer | | 2,250 | 680 | 1.28 | 1.15 | 1.43 | 441 | 31 | 1.11 | 0.72 | 1.71 |
| | <2 | 435 | 157 | 1.88 | 1.50 | 2.35 | 71 | 4 | 1.27 | 0.44 | 3.67 |
| | 2-5 | 478 | 131 | 1.29 | 1.02 | 1.62 | 103 | 8 | 1.25 | 0.54 | 2.89 |
| | >5 | 1,337 | 392 | 1.11 | 0.97 | 1.28 | 267 | 19 | 1.02 | 0.60 | 1.73 |
| **No infection, 1 dose** | | | | | | | | | | | |
| HR for pop no cancer | | 1,172 | 72 | 0.40 | 0.30 | 0.52 | 2,281 | 26 | 1.04 | 0.68 | 1.61 |
| HR for pop with cancer | | 60 | 22 | 1.08 | 0.60 | 1.91 | 37 | 2 | 0.77 | 0.15 | 3.85 |
| | <2 | 16 | 6 | 1.55 | 0.54 | 4.50 | 10 | 0 | - | - | - |
| | 2-5 | 10 | 2 | 0.24 | 0.05 | 1.18 | 7 | 0 | - | - | - |
| | >5 | 34 | 14 | 1.40 | 0.65 | 3.01 | 20 | 2 | 1.59 | 0.29 | 8.81 |
| **No infection, 2 doses** | | | | | | | | | | | |
| HR for pop no cancer | | 3,971 | 142 | 0.27 | 0.23 | 0.33 | 30,536 | 154 | 1.59 | 0.29 | 8.81 |
| HR for pop with cancer | | 237 | 39 | 0.57 | 0.39 | 0.82 | 581 | 38 | 1.09 | 0.74 | 1.58 |
| | <2 | 40 | 7 | 0.77 | 0.31 | 1.92 | 141 | 18 | 3.24 | 1.86 | 5.62 |
| | 2-5 | 54 | 7 | 0.51 | 0.22 | 1.21 | 88 | 4 | 0.57 | 0.19 | 1.72 |
| | >5 | 143 | 25 | 0.54 | 0.34 | 0.86 | 352 | 16 | 0.57 | 0.19 | 1.72 |
| **No infection, 3 or more doses** | | | | | | | | | | | |
| HR for pop no cancer | | 228 | 21 | 0.35 | 0.22 | 0.57 | 61,709 | 821 | 0.24 | 0.21 | 0.28 |
| HR for pop with cancer | | 34 | 6 | 0.55 | 0.21 | 1.42 | 5,059 | 288 | 0.43 | 0.34 | 0.53 |
| | <2 | 8 | 1 | 0.48 | 0.05 | 4.31 | 759 | 57 | 0.79 | 0.57 | 1.10 |
| | 2-5 | 8 | 2 | 0.71 | 0.13 | 3.88 | 1,105 | 60 | 0.46 | 0.33 | 0.65 |
| | >5 | 18 | 3 | 0.50 | 0.13 | 1.91 | 3,195 | 171 | 0.34 | 0.27 | 0.44 |
| **Infection, no vaccine** | | | | | | | | | | | |
| HR for pop no cancer | | 128 | 10 | 0.78 | 0.37 | 1.67 | 3,923 | 15 | 0.34 | 0.20 | 0.59 |
| HR for pop with cancer | | 8 | 2 | 0.38 | 0.07 | 2.19 | 74 | 1 | 0.32 | 0.04 | 2.39 |
| | <2 | 1 | 0 | - | - | - | 16 | 0 | - | - | - |
| | 2-5 | 3 | 0 | - | - | - | 17 | 0 | - | - | - |
| | >5 | 4 | 2 | 1.43 | 0.18 | 11.49 | 41 | 1 | 0.73 | 0.10 | 5.51 |
| **Infection, 1 dose** | | | | | | | | | | | |
| HR for pop no cancer | | 21 | 1 | 0.57 | 0.06 | 5.57 | 1,527 | 12 | 0.66 | 0.36 | 1.22 |
| HR for pop with cancer | | 1 | 0 | - | - | - | 34 | 0 | - | - | - |
| | <2 | 0 | 0 | - | - | - | 6 | 0 | - | - | - |
| | 2-5 | 0 | 0 | - | - | - | 8 | 0 | - | - | - |
| | >5 | 1 | 0 | - | - | - | 20 | 0 | - | - | - |
| **Infection, 2 doses** | | | | | | | | | | | |
| HR for pop no cancer | | 14 | 0 | - | - | - | 3,620 | 25 | 0.66 | 0.36 | 1.22 |
| HR for pop with cancer | | 1 | 0 | - | - | - | 158 | 9 | 0.50 | 0.23 | 1.08 |
| | <2 | 0 | 0 | - | - | - | 21 | 2 | 1.23 | 0.25 | 6.13 |
| | 2-5 | 0 | 0 | - | - | - | 34 | 1 | 0.42 | 0.05 | 3.55 |
| | >5 | 1 | 0 | - | - | - | 103 | 6 | 0.41 | 0.16 | 1.05 |
| **Infection, 3 or more doses** | | | | | | | | | | | |
| HR for pop no cancer | | 1 | 0 | - | - | - | 2,353 | 24 | 0.16 | 0.10 | 0.25 |
| HR for pop with cancer | | 0 | 0 | - | - | - | 160 | 5 | 0.21 | 0.08 | 0.57 |
| | <2 | 0 | 0 | - | - | - | 29 | 4 | 2.25 | 0.70 | 7.23 |
| | 2-5 | 0 | 0 | - | - | - | 32 | 0 | - | - | - |
| | >5 | 0 | 0 | - | - | - | 99 | 1 | 0.04 | 0.01 | 0.34 |

**Table S7.** Risk of SARS-CoV-2 infection for population with cancer by immunisation status adjusted for sex, age and Charlson Comorbidity Index in the pre-Omicron and Omicron BA.1 periods, Reggio Emilia province, Italy, 20 February 2020–30 September 2022.

| | | Risk of SARS-CoV-2 infection | | | | | | | | | | |
|---|---|---|---|---|---|---|---|---|---|---|---|---|
| | | Period of infection from Feb 20, 2020 to Dec 20, 2021 | | | | | Period of infection from Jan 01, 2022 to Sep 30, 2022 | | | | | |
| | | Persons-days | Infections | HR | 95% IC | | Persons-days | Infections | HR | 95% IC | | |
| **No infection, no vaccine** | | | | | | | | | | | | |
| HR for pop with cancer | | 11,841,178 | 2,210 | 1 | | | 321,217 | 435 | 1 | | | |
| | <2 | 1,504,078 | 429 | 1.54 | 1.38 | 1.71 | 25,634 | 70 | 1.70 | 1.30 | 2.23 | |
| | 2-5 | 2,540,316 | 470 | 0.96 | 0.86 | 1.06 | 71,183 | 101 | 1.04 | 0.82 | 1.31 | |
| | >5 | 7,796,784 | 1,311 | 0.80 | 0.73 | 0.87 | 224,400 | 264 | 0.75 | 0.61 | 0.91 | |
| **No infection, 1 dose** | | | | | | | | | | | | |
| HR for pop with cancer | | 918,722 | 80 | 0.60 | 0.46 | 0.77 | 23,883 | 36 | 0.79 | 0.56 | 1.11 | |
| | <2 | 141,877 | 16 | 0.71 | 0.42 | 1.17 | 4,813 | 9 | 0.96 | 0.49 | 1.86 | |
| | 2-5 | 171,583 | 14 | 0.50 | 0.29 | 0.86 | 4,147 | 8 | 1.02 | 0.51 | 2.01 | |
| | >5 | 605,262 | 50 | 0.58 | 0.42 | 0.79 | 14,923 | 19 | 0.67 | 0.42 | 1.06 | |
| **No infection, 2 doses** | | | | | | | | | | | | |
| HR for pop with cancer | | 4,736,273 | 257 | 0.37 | 0.30 | 0.45 | 331,742 | 588 | 0.99 | 0.87 | 1.13 | |
| | <2 | 690,189 | 46 | 0.48 | 0.34 | 0.68 | 64,425 | 143 | 1.13 | 0.93 | 1.38 | |
| | 2-5 | 979,014 | 58 | 0.43 | 0.32 | 0.59 | 60,692 | 89 | 0.81 | 0.64 | 1.02 | |
| | >5 | 3,067,070 | 153 | 0.34 | 0.27 | 0.42 | 206,625 | 356 | 1.00 | 0.86 | 1.15 | |
| **No infection, 3 or more doses** | | | | | | | | | | | | |
| HR for pop with cancer | | 567,032 | 34 | 0.09 | 0.06 | 0.15 | 5,571,101 | 5,059 | 0.75 | 0.67 | 0.83 | |
| | <2 | 75,587 | 8 | 0.18 | 0.08 | 0.38 | 648,840 | 759 | 0.97 | 0.86 | 1.09 | |
| | 2-5 | 119,224 | 8 | 0.10 | 0.05 | 0.20 | 1,126,850 | 1,105 | 0.79 | 0.71 | 0.89 | |
| | >5 | 372,221 | 18 | 0.08 | 0.04 | 0.13 | 3,795,411 | 3,195 | 0.70 | 0.63 | 0.78 | |
| **Infection, no vaccine** | | | | | | | | | | | | |
| HR for pop with cancer | | 222,370 | 8 | 0.18 | 0.09 | 0.36 | 88,452 | 73 | 0.86 | 0.66 | 1.13 | |
| | <2 | 37,821 | 1 | 0.13 | 0.02 | 0.96 | 12,696 | 16 | 1.22 | 0.76 | 1.97 | |
| | 2-5 | 46,579 | 3 | 0.31 | 0.10 | 0.96 | 18,195 | 17 | 0.96 | 0.59 | 1.58 | |
| | >5 | 137,970 | 4 | 0.15 | 0.05 | 0.39 | 57,561 | 40 | 0.73 | 0.52 | 1.03 | |
| **Infection, 1 dose** | | | | | | | | | | | | |
| HR for pop with cancer | | 228,024 | 1 | 0.03 | 0.00 | 0.20 | 32,247 | 34 | 0.50 | 0.35 | 0.71 | |
| | <2 | 31,728 | 0 | - | - | - | 5,115 | 6 | 0.53 | 0.25 | 1.14 | |
| | 2-5 | 54,258 | 0 | - | - | - | 5,835 | 7 | 0.48 | 0.23 | 1.02 | |
| | >5 | 142,038 | 1 | 0.05 | 0.01 | 0.33 | 21,297 | 21 | 0.49 | 0.31 | 0.75 | |
| **Infection, 2 doses** | | | | | | | | | | | | |
| HR for pop with cancer | | 141,443 | 1 | 0.03 | 0.00 | 0.21 | 352,240 | 159 | 0.38 | 0.32 | 0.46 | |
| | <2 | 25,339 | 0 | - | - | - | 46,563 | 21 | 0.38 | 0.25 | 0.58 | |
| | 2-5 | 28,187 | 0 | - | - | - | 79,741 | 35 | 0.37 | 0.26 | 0.51 | |
| | >5 | 87,917 | 1 | 0.05 | 0.01 | 0.33 | 225,936 | 103 | 0.40 | 0.33 | 0.50 | |
| **Infection, 3 or more doses** | | | | | | | | | | | | |
| HR for pop with cancer | | 10,716 | 0 | - | - | - | 566,147 | 160 | 0.36 | 0.30 | 0.44 | |
| | <2 | 1,977 | 0 | - | - | - | 71,946 | 29 | 0.48 | 0.33 | 0.69 | |
| | 2-5 | 2,497 | 0 | - | - | - | 131,307 | 32 | 0.33 | 0.23 | 0.47 | |
| | >5 | 6,242 | 0 | - | - | - | 362,894 | 99 | 0.37 | 0.30 | 0.46 | |

**Table S8.** Risk of severe disease and death from COVID-19 for population with cancer by immunisation status adjusted for sex, age and Charlson Comorbidity Index in the pre-Omicron and Omicron BA.1 periods, Reggio Emilia province, Italy, 20 February 2020–30 September 2022.

| | Risk of severe disease and death from COVID-19 | | | | | | | | | |
|---|---|---|---|---|---|---|---|---|---|---|
| | Period of infection from Feb 20, 2020 to Dec 20, 2021 | | | | | Period of infection from Jan 01, 2022 to Sep 30, 2022 | | | | |
| | n | Events | OR | 95% IC | | n | Events | OR | 95% IC | |
| **No infection, no vaccine** | | | | | | | | | | |
| HR for pop with cancer | 2,210 | 665 | 1 | | | 435 | 31 | 1 | | |
| <2 | 429 | 153 | 1.54 | 1.20 | 1.97 | 70 | 4 | 1.05 | 0.33 | 3.40 |
| 2-5 | 470 | 128 | 0.99 | 0.77 | 1.28 | 101 | 8 | 1.33 | 0.51 | 3.49 |
| >5 | 1,311 | 384 | 0.75 | 0.61 | 0.92 | 264 | 19 | 0.78 | 0.33 | 1.84 |
| **No infection, 1 dose** | | | | | | | | | | |
| HR for pop with cancer | 80 | 30 | 1.03 | 0.62 | 1.70 | 36 | 2 | 0.85 | 0.18 | 4.08 |
| <2 | 16 | 6 | 1.31 | 0.44 | 3.87 | 9 | 0 | - | - | - |
| 2-5 | 14 | 5 | 1.13 | 0.32 | 3.95 | 8 | 0 | - | - | - |
| >5 | 50 | 19 | 0.93 | 0.49 | 1.74 | 19 | 2 | 1.62 | 0.31 | 8.51 |
| **No infection, 2 doses** | | | | | | | | | | |
| HR for pop with cancer | 257 | 46 | 0.49 | 0.34 | 0.69 | 588 | 38 | 0.88 | 0.52 | 1.47 |
| <2 | 46 | 11 | 0.87 | 0.41 | 1.83 | 143 | 18 | 2.48 | 1.26 | 4.89 |
| 2-5 | 58 | 7 | 0.32 | 0.14 | 0.73 | 89 | 4 | 0.53 | 0.16 | 1.76 |
| >5 | 153 | 28 | 0.46 | 0.29 | 0.72 | 356 | 16 | 0.55 | 0.29 | 1.07 |
| **No infection, 3 or more doses** | | | | | | | | | | |
| HR for pop with cancer | 34 | 6 | 0.42 | 0.17 | 1.09 | 5,059 | 288 | 0.37 | 0.24 | 0.56 |
| <2 | 8 | 1 | 0.35 | 0.04 | 3.19 | 759 | 57 | 0.68 | 0.41 | 1.12 |
| 2-5 | 8 | 2 | 0.54 | 0.10 | 2.90 | 1,105 | 60 | 0.40 | 0.24 | 0.66 |
| >5 | 18 | 3 | 0.39 | 0.10 | 1.50 | 3,195 | 171 | 0.28 | 0.18 | 0.45 |
| **Infection, no vaccine** | | | | | | | | | | |
| HR for pop with cancer | 8 | 2 | 0.37 | 0.07 | 1.98 | 73 | 1 | 0.28 | 0.04 | 2.16 |
| <2 | 1 | 0 | - | - | - | 16 | 0 | - | - | - |
| 2-5 | 3 | 0 | - | - | - | 17 | 0 | - | - | - |
| >5 | 4 | 2 | 1.21 | 0.15 | 9.67 | 40 | 1 | 0.56 | 0.07 | 4.40 |
| **Infection, 1 dose** | | | | | | | | | | |
| HR for pop with cancer | 1 | 0 | - | - | - | 34 | 0 | - | - | - |
| <2 | 0 | 0 | - | - | - | 6 | 0 | - | - | - |
| 2-5 | 0 | 0 | - | - | - | 7 | 0 | - | - | - |
| >5 | 1 | 0 | - | - | - | 21 | 0 | - | - | - |
| **Infection, 2 doses** | | | | | | | | | | |
| HR for pop with cancer | 1 | 0 | - | - | - | 159 | 9 | 0.46 | 0.19 | 1.10 |
| <2 | 0 | 0 | - | - | - | 21 | 2 | 1.16 | 0.22 | 5.94 |
| 2-5 | 0 | 0 | - | - | - | 35 | 1 | 0.30 | 0.03 | 3.10 |
| >5 | 1 | 0 | - | - | - | 103 | 6 | 0.40 | 0.14 | 1.12 |
| **Infection, 3 or more doses** | | | | | | | | | | |
| HR for pop with cancer | 0 | 0 | - | - | - | 160 | 5 | 0.22 | 0.08 | 0.63 |
| <2 | 0 | 0 | - | - | - | 29 | 4 | 2.03 | 0.59 | 7.01 |
| 2-5 | 0 | 0 | - | - | - | 32 | 0 | - | - | - |
| >5 | 0 | 0 | - | - | - | 99 | 1 | 0.04 | 0.00 | 0.34 |
